# Confirmed *Mycoplasma pneumoniae* Endocarditis

**DOI:** 10.3201/eid1410.080157

**Published:** 2008-10

**Authors:** Juan Pablo Scapini, Luis Pedro Flynn, Silvia Sciacaluga, Lorena Morales, María Estela Cadario

**Affiliations:** Facultad de Ciencias Médicas Universidad Nacional de Rosario, Santa Fe, Argentina (J.P. Scapini); Sanatorio de Niños Rosario, Santa Fe, (L.P. Flynn S. Sciacaluga, L. Morales); Instituto Nacional de Enfermedades Infecciosas ANLIS “Dr Carlos Malbrán,” Buenos Aires, Argentina (M.E. Cadario)

**Keywords:** *Mycoplasma*, endocarditis, bacteremia, letter

**To the Editor:** In Rosario, Argentina, during June 2005, a 15-year-old boy was hospitalized because of a 2-month history of fever. The patient had no history of cardiac disease or intravenous drug use. The results of the physical examination and the laboratory tests were within normal limits, except for an increased leukocyte count (14,000/µL) with 68% neutrophils.

Transesophagic echocardiography showed mural vegetation on the right ventricle (30 mm × 20 mm) with no valve involvement. The patient was empirically treated with penicillin, gentamicin, and ceftriaxone. After treatment failed to produce a response, blood was submitted for culture for mycobacteria, brucellae, bartonellae, molds, and yeasts. BacT/ALERT bottles (bioMérieux, Durham, NC, USA), Hemoline performance biphasic medium (bioMérieux, Marcy L’Etoile, France), lysis centrifugation, and homemade culture broth were used. All culture results were negative. Results of PCR performed on serum for *Actinobacillus actinomycetemcomitans* were also negative. Because only the first samples were obtained before antimicrobial drug administration, a false-negative result was suspected. The patient underwent surgery for pulmonary microembolisms, and the vegetation was removed 4 weeks after drug treatment had started. The histologic appearance of the vegetation was consistent with infectious endocarditis, but the culture result was negative.

After 6 weeks of treatment, the patient was discharged from the hospital; however, 10 days after discharge he again became febrile and was readmitted to the hospital. The vegetation was again found. On this second admission, all cultures were performed before administration of antimicrobial drugs, and several types of culture media were used. In the absence of any growth by day 6, the patient’s serum was screened for antibodies to *Mycoplasma pneumoniae, Chlamydia pneumoniae, and Bartonella henselae*. Serologic tests for immunoglobulin (Ig) G and IgM were conducted by indirect immunofluorescence assay (slides from Bion; Des Plaines, IL, USA) and fluorescein-labeled anti-human IgG and IgM (bioMérieux). For the IgM assay, the serum was pretreated with IgG/RF stripper (The Binding Site Ltd., Birmingham, UK). The titers for *M. pneumoniae* IgG and IgM antibodies were 2,048 and 160, respectively. Blood cultures were then subcultured in homemade Hayflick medium. These samples were incubated in 5% CO_2_ in a 37°C incubator and examined 2×/week for typical *M. pneumoniae* colonies.

After 9 days of incubation, Hayflick agar plates inoculated with aliquots taken from homemade blood culture bottles (beef extract 5 g, yeast extract 5 g, peptone 10 g, glucose 2 g, NaCl 5 g, Na_2_HPO_4_ 2.5 g, sodium heparin 10,000 U, distilled water to 1,000 mL, pH 7.6) showed colonies consistent with *M. pneumoniae.* No isolates were recovered from commercial blood culture bottles.

Result of hemolysis test with sheep blood was positive. The isolate was definitively identified as *M. pneumoniae* after P1 cytadhesin gene amplification by nested PCR, with primers P1-40, P1-178, P1-285, and P1-331 (*1*).

After mycoplasma were was isolated, intravenous clarithromycin was added to ceftriaxone; the ceftriaxone was discontinued 1 week later. The patient’s clinical condition improved, and he was discharged 3 weeks after bacteriologic diagnosis with a treatment regimen of oral levofloxacin. After 6 months of treatment, the vegetation was reduced with no evidence of calcification.

*Mycoplasma* spp. have rarely been associated with endocarditis; until 2007, reports of only 8 cases had been published (*2*–*8*). The patient described herein had no underlying medical problems or immunodeficiency. Results of lymphocyte subsets, immunoglobulin titers, response to tetanus toxoid, and pneumococcal capsular polysaccharide were within reference ranges.

Cases of culture-negative endocarditis are not routinely investigated for mycoplasmas; however, the role of these microorganisms as a cause of endocarditis might be underestimated. *Mycoplasma* spp. cannot be detected by Gram stain and are difficult to isolate in bacteriologic culture media. Commercial blood culture broths that use sodium polyanetholsulfonate as an anticlotting agent are not suitable for growing these microorganisms (*9*). Other diagnostic approaches include the detection of specific DNA sequences or the use of broad-range eubacterial primers in cardiac tissue (*6*). In the patient reported here, the clinical sample (vegetation) was not available for diagnostic *M. pneumoniae* gene amplification. We failed to detect *M. pneumoniae* by PCR-mediated gene amplification directly from whole blood and plasma. Theoretically, specific PCR should be more sensitive than culture, as shown in respiratory specimens, but to date attempts to detect *M. pneumoniae* in blood by PCR have not been successful. The bacterial load in blood may have been too low to detect the amplified product by ethidium bromide–stained gel electrophoresis. The larger volume of blood used and the preincubation in broth with yeast extract for 7 days could have improved the recovery by culture. Another cause of reduced PCR sensitivity may have been the use of frozen samples.

This case of endocarditis caused by *M. pneumoniae* was confirmed by culture and occurred in a patient with no previous heart disease. Further studies are needed to evaluate the real incidence of *M. pneumoniae* as a cause of endocarditis as well as the occurrence of mycoplasma bacteremia in the absence of underlying infection of the endocardium.
